# Serotyping of *Toxoplasma gondii* Infection Using Peptide Membrane Arrays

**DOI:** 10.3389/fcimb.2019.00408

**Published:** 2019-11-29

**Authors:** David Arranz-Solís, Cynthia Cordeiro, Lucy H. Young, Marie Laure Dardé, Alessandra G. Commodaro, Michael E. Grigg, Jeroen P. J. Saeij

**Affiliations:** ^1^Department of Pathology, Microbiology and Immunology, School of Veterinary Medicine, University of California, Davis, Davis, CA, United States; ^2^Department of Ophthalmology, Massachusetts Eye and Ear Infirmary, Boston, MA, United States; ^3^Biology Department, Massachusetts Institute of Technology, Cambridge, MA, United States; ^4^Faculty of Medicine, Parasitologie-Mycologie, UMR INSERM 1094, National Reference Center and Biological Resource Center for Toxoplasmosis, CHU Dupuytren 2, Limoges, France; ^5^Molecular Parasitology Section, Laboratory of Parasitic Diseases, National Institute of Allergy and Infectious Diseases, National Institutes of Health, Bethesda, MD, United States

**Keywords:** *Toxoplasma*, serotyping, strain type, dense granule, peptide, microarrays

## Abstract

The intracellular parasite *Toxoplasma gondii* can cause chronic infections in most warm-blooded animals, including humans. In the USA, strains belonging to four different *Toxoplasma* clonal lineages (types 1, 2, 3, and 12) are commonly isolated, whereas strains not belonging to these lineages are predominant in other continents such as South America. Strain type plays a pivotal role in determining the severity of *Toxoplasma* infection. Therefore, it is epidemiologically relevant to develop a non-invasive and inexpensive method for determining the strain type in *Toxoplasma* infections and to correlate the genotype with disease outcome. Serological typing is based on the fact that many host antibodies are raised against immunodominant parasite proteins that are highly polymorphic between strains. However, current serological assays can only reliably distinguish type 2 from non-type 2 infections. To improve these assays, mouse, rabbit, and human infection serum were reacted against 950 peptides from 62 different polymorphic *Toxoplasma* proteins by using cellulose membrane peptide arrays. This allowed us to identify the most antigenic peptides and to pinpoint the most relevant polymorphisms that determine strain specificity. Our results confirm the utility of previously described peptides and identify novel peptides that improve and increase the specificity of the assay. In addition, a large number of novel proteins showed potential to be used for *Toxoplasma* diagnosis. Among these, peptides derived from several rhoptry, dense granule, and surface proteins represented promising candidates that may be used in future experiments to improve *Toxoplasma* serotyping. Moreover, a redesigned version of the published GRA7 typing peptide performed better and specifically distinguished type 3 from non-type 3 infections in sera from mice, rabbits, and humans.

## Introduction

*Toxoplasma gondii* is a ubiquitous obligate intracellular protozoan parasite that can infect virtually all warm-blooded animals, including humans (Dabritz and Conrad, 2010). Although infection is usually asymptomatic, it can also cause ocular toxoplasmosis (OT) in immunocompetent individuals, encephalitis in immunocompromised individuals and abortion, birth defects or congenital OT in newborns from pregnant women infected for the first time (Furtado et al., 2011). Indeed, the progression and severity of toxoplasmosis depends on a combination of host and parasite factors. The *Toxoplasma* strain type that causes infection is one of the key factors known to influence the outcome of the disease (Boothroyd and Grigg, 2002; Ajzenberg et al., 2010; Fekkar et al., 2011). In the USA, strains belonging to four different *Toxoplasma* clonal lineages (types 1, 2, 3, and 12) are commonly isolated from animals, whereas strains not belonging to these lineages are predominant in other continents such as South America (Herrmann et al., 2012; Su et al., 2012). The latter are more virulent in mice compared to isolates from North America, Europe, North Africa and Asia (Shwab et al., 2016). Likewise, severe systemic toxoplasmosis resulting in death of immune competent people infected with parasite strains from South America have been reported (Carme et al., 2009; Hamilton et al., 2019). By contrast, type 2 strains are the most prevalent cause of human toxoplasmosis in Europe and North America but are less often associated with the most severe clinical cases. For example, in both congenital infection and in immunosuppressed patients in the USA and Europe, type 1 and other strains not common to these regions are more likely to be found infecting immunocompetent individuals suffering from severe, atypical OT (Grigg et al., 2002; Shobab et al., 2013), or are disproportionately associated with severe congenital toxoplasmosis (Howe et al., 1997; Fuentes et al., 2001; Carme et al., 2002, 2009; McLeod et al., 2012; Hutson et al., 2015). Nevertheless, caution is needed when interpreting these reports, as most of these studies relied on only a few molecular markers or alleles to identify the strain type by PCR-restriction fragment length polymorphism (RFLP) or microsatellite markers, and thus might be misclassified (Lorenzi et al., 2016).

It is increasingly appreciated that if the strain genotype causing infection is known, then treatment could be matched to the specifics of the infection; for example patients infected with virulent strains that have not yet developed OT could be identified, and treatment options altered, such as more frequent eye exams or prophylactic treatment to improve prognostic outcomes (Arantes et al., 2015). A previous work showed that OT patients infected with non-type 2 strains have a higher chance of recurrent disease (Shobab et al., 2013), hence long-term treatment could be recommended to patients infected with these strains. Furthermore, once an association between *Toxoplasma* strain and disease phenotype is established, experiments can be designed to determine the molecular basis for the increased virulence of the strain, which might ultimately lead to novel therapies. It is therefore epidemiologically relevant to determine the types dominating in particular regions and to investigate if there are correlations between strain type and severity of toxoplasmosis.

To enable large-scale investigations into the influence of parasite genotype on the severity of disease, an assay that identifies the strain infecting a patient is required. However, the ability to genotype the infecting strain is often limited by insufficient parasite DNA present in a patient's sample (e.g., amniocentesis or vitreous fluid) (Lorenzi et al., 2016). Moreover, parasites can only be obtained from symptomatic individuals in very low amounts through difficult and risky biopsies. Hence, what is needed is a rapid, highly sensitive, and non-invasive means of identifying strain type in any disease state. In this sense, and in contrast to the DNA-dependent techniques, serotyping allows not only the inclusion of clinical, but also subclinical cases. This is extremely important not only to provide a means for early detection of toxoplasmosis before clinical signs are established, but it also identifies the strain type infecting asymptomatic individuals, which may provide new insight into *Toxoplasma* epidemiology.

This so-called serological typing is based on the fact that many host antibodies are raised against parasite proteins that are highly polymorphic among distinct strains. Furthermore, *Toxoplasma* stimulates a strong and persistent humoral immune response in every host: antibodies to parasite proteins remain at high titers for the life of the host and are present in patients regardless of the clinical manifestations. In the last two decades, some advances have been made in the development of these tests to determine the strain from infected hosts by using polymorphic parasite peptides (Dard et al., 2016). By coating ELISA plates, nitrocellulose membranes, or glass slides with these polymorphic peptides and monitoring the reactivity of the serum, a prediction of the infecting strain type can be made. Several studies have demonstrated the usefulness of such tests in different species (Kong et al., 2003; Nowakowska et al., 2006; Peyron et al., 2006; Morisset et al., 2008; Vaudaux et al., 2010; Maksimov et al., 2012a,b, 2013, 2018; McLeod et al., 2012; Shobab et al., 2013). However, although some attempts have been made to differentiate the three archetypal strains (type 1, 2, and 3), unfortunately, at the moment these methods can only reliably distinguish type 2 from non-type 2 strains, typically type 1 or 3 (Kong et al., 2003; Sousa et al., 2009; Xiao et al., 2009; Maksimov et al., 2012b, 2018; McLeod et al., 2012).

Since it is extremely unlikely that a single or only a few peptides can distinguish strain types reliably, the identification of a large number of peptides that allow for the detection of the total diversity of existing strains would be a major advancement in *Toxoplasma* serotyping. In the present study, we validated previously described peptides, and identified new *Toxoplasma* antigenic peptides that could be used to discriminate between infections caused by different strain types based on whole genome comparisons of the 64 *Toxoplasma* strains available in ToxoDB (http://ToxoDB.org; Gajria et al., 2008). In addition to these polymorphic peptides, we also screened the antigenicity and specificity of peptide sequences from genes expressed in some strains but absent in others. It is worth mentioning that peptides, regardless of the strain genotype, are able to provide a specific mark; hence they may recognize clonal as well as recombinant strain types as long as they possess the specific type sequence of the peptide. Therefore, by increasing the number of peptides that can be recognized by an individual serum, in the future a specific “fingerprint” for each strain might be defined, similar to current RFLP methods. This, in turn, will allow large studies to be conducted that correlate peptide epitopes as markers for infecting strains with specific disease outcomes, which may help to better understand how strain type influences disease outcome.

## Methods

### Toxoplasma *in vitro* Culture

*Toxoplasma* strains used for animal infections were routinely maintained on Human Foreskin Fibroblast (HFF) monolayers in Dulbecco's modified Eagle's medium (DMEM; Life Technologies) supplemented with 1% fetal bovine serum (FBS; Omega Scientific), 4.5 g/liter D-glucose, L-glutamine and antibiotics as previously described (Jensen et al., 2015).

### Ethics Statement and Mice Infection

All mouse work was performed in accordance with the recommendations in the Guide to the Care and Use of Laboratory Animals (104) of the National Institutes of Health. The MIT Committee on Animal Care (assurance no. A-3125-01) and the Animal Care and Use Committee of the Intramural Research Program of NIAID (Animal Study Protocol LPD22E) approved all protocols, and all efforts were made to minimize unnecessary distress to the animals. Human samples were used according to the Committee on the Use of Humans as Experimental Subjects (COUHES) application No. 0808002869.

### Serum Samples

Different serum samples from mice, rabbits and human patients were used in the present study.

#### Mouse Samples

Sera from mice chronically infected with type 1 RH; type 2 ME49, Prugniaud -Pru-, FORT, WIL; and type 3 CEP, VEG, and C56 were used (Kong et al., 2003). Depending on the strain, infection was carried out by intraperitoneal injection of tachyzoites or oral gavage of 3–10 cysts in a volume of 0.2 ml. Animals were checked every day to detect signs of illness such as rough hair coat, apathy, or weight loss. Depending on the strain, sulfadiazine, alone, or in combination with pyrimethamine, was added to drinking water (0.4 and 0.2 mg/ml), either at the time of infection or upon detection of clinical signs, to control the acute stage. The sera of infected mice were collected at different time points according to the severity of infection caused by specific strains.

#### Rabbit Samples

Serum samples from rabbits infected with RH (type 1), ME49 (type 2), VEG (type 3), and WIL (type 2) strains were a kind gift from Dr. Steve Parmley, Palo Alto Medical Foundation.

#### Human Samples

Serum samples from infected patients genotyped either in France (Center national de reference-Toxoplasmose Limoges France) (Sousa et al., 2008, 2009) or in the United States (de-la-Torre et al., 2013; Shobab et al., 2013) were used, as well as samples from healthy infected individuals and OT patients from Brazil but living in USA. Table 1 has all the human serum information.

**Table 1:** Human serum samples employed in peptide arrays and ELISA.

**Name**	**Type**	**Country**	**Infection origin**	**Array number**	**References**
Donor 1	NA	Brazil	Unknown	2, 4	This study
Donor 2	NA	Brazil	Unknown	4	This study
Patient 1	NA	Brazil	Unknown	2, 4	This study
Patient 2	NA	Brazil	Unknown	2, 4	This study
Patient 3	NA	Brazil	Unknown	4	This study
Patient 4	NA	Brazil	Unknown	4	This study
Patient 5	NA	Brazil	Unknown	4	This study
D83	1	USA	Ocular	4, 5	This study
NS	2	USA	Lab accident	4	This study
TIL	3	USA	Lab accident	4, 5	This study
IPP005-URB B (IppUrbB)	16	France	Imported horse-meat consumption (reinfection)	5	Elbez-Rubinstein et al., 2009; Lorenzi et al., 2016
TyI FAJI	1	France	Congenital	5	Ajzenberg et al., 2002
TyII Fr2a	2	France	Unknown	5	Sousa et al., 2008
TyII Fr4b	2	France	Unknown	5	Sousa et al., 2008
TyII Fr17b	2	France	Unknown	5	Sousa et al., 2008
TyIII Fr18a (NED)	3	France	Congenital	5	Sousa et al., 2008, 2009
TyIII Fr19a	3	France	Unknown	5	Sousa et al., 2008, 2009
TyIII Fr20a (TOU-FEU)	3	France	Unknown	5	Sousa et al., 2008, 2009
MAS	4	France	Imported horse-meat consumption	5	Gallego et al., 2004; Pomares et al., 2011
TOU021-ALI (TOU-ALI)	4	Reunion Island (France)	Unknown	5	Gallego et al., 2004; Sousa et al., 2008; Su et al., 2012
GUY014-TER (GUY-TER)	Atypical	Suriname	Unknown	5	Sousa et al., 2008, 2009
VAND	10	French Guiana (France)	Unknown	5	Gallego et al., 2004; Lorenzi et al., 2016

*NA, not available*.

### Selection of Genes and Epitopes

The complete genome sequences of multiple *Toxoplasma* strains are available in ToxoDB (http://ToxoDB.org; Gajria et al., 2008). Because *Toxoplasma* polymorphisms are mostly bi-allelic (i.e., at each SNP there are often only two polymorphic nucleotides) (Grigg et al., 2001), we searched for polymorphic proteins with signal peptides (secreted or surface proteins are more likely to be antigenic) when comparing *Toxoplasma* strains 1 vs. 2, 2 vs. 3 and 1 vs. 3. Fifty polymorphic genes were selected from this comparison, including the virulence factors ROP5, ROP18, ROP16, and GRA15, as well as other rhoptry (ROP8 and ROP20) and dense granule proteins (GRA3, GRA5, GRA6, and GRA7) (Supplemental Table 1). The type 1, 2, and 3 sequences from the selected proteins were downloaded from ToxoDB and protein alignments were made using ClustalX (Thompson et al., 1997). Regions in those alignments that were polymorphic and predicted to be antigenic (see below) were considered for peptide synthesis. Besides polymorphic peptides, we picked peptides derived from proteins that are expressed in one strain but not in another strain. These candidates came from genes that were previously found to be differentially expressed between different strains using *Toxoplasma* RNAseq (Melo et al., 2013), along with information from other groups on gene expression levels in type 1, 2, and 3 strains that is available on ToxoDB. The best example of this is the *ROP18* virulence gene, which is not expressed in type 3 but highly expressed in types 1 and 2 (Saeij et al., 2006). In total we tested peptides from 62 *Toxoplasma* genes, present on each of its 14 different chromosomes. Peptides to be synthesized were selected on the basis of the following criteria: focusing on short regions of typically 8–12 amino acids close to the N- or C-terminus, hydrophilicity (Kyte-Doolittle plots), presence of β-turns (ChouFasman), and good antigenic and surface probability indexes (Jameson-Wolf and Emini's surface plots) were considered. To predict potential antigenic peptides, we used computer algorithms (available at http://ca.expasy.org/tools/) that predict protein hydrophilicity and tendency to form turns. In total, 950 peptides derived from these proteins, as well as 53 control peptides (see below), were used in the present study (Supplemental Table 1).

### Peptide Arrays

To be able to screen large numbers of peptides for their antigenicity and the ability to discriminate between distinct *Toxoplasma* strains, an ABIMED peptide arrayer system (MIT Biopolymer facility) was used to construct cellulose peptide arrays each containing 600 peptide spots. This system consists of a computer-controlled Gilson diluter and XYZ liquid handling robot which allows the deposition on amino-PEG cellulose membranes of individual activated amino acids resulting in peptide formation. Each spot contains 20 nmol starting peptide. As an internal control we included an alanine scan of the haemagglutinin (HA) epitope (YPYDVPDYA) for which specific monoclonal antibodies are available. Polymorphic peptides from GRA5, GRA6, and GRA7, known to be able to discriminate between infections with different strain types (Kong et al., 2003), served as positive controls, while peptides from other pathogens were included as negative controls (Reineke et al., 2002; Weiser et al., 2005; Albert et al., 2008) (Supplemental Table 1).

A total of 5 different arrays were carried out, containing 597, 300, 100, 120, and 40 peptides, respectively. For the first three arrays, serum samples from chronically infected mice infected with type 1, 2, or 3 strains were used. In addition, sera from experimentally infected rabbits were also used in array 3. Finally, human samples from healthy donors that tested positive for *Toxoplasma* were used in array 2, whereas serum samples from OT patients, as well as from infected patients with a genotyped *Toxoplasma* strain, were used in arrays 4 and 5 (Table 1). Serum samples from uninfected healthy mice and humans served as negative controls. The first two arrays were used to find peptides that were antigenic, while the other three were used to refine promising peptides using a suite of type-specific serum samples from human patients.

The protocol to detect antibody binding to specific peptides was similar to a traditional Western blot. In short, the peptide array was placed in a container and blocked overnight with 5% BSA, 5% dry milk, 0.1% Tween-20 PBS. Subsequently, diluted serum (1/100–400, adjusted depending on the titer) was added and after 2 h incubation the blot washed (0.1% Tween-20 PBS). Then, an HRP-conjugated secondary antibody (goat α-mouse, α-human or α-rabbit, 1/10,000, Sigma) was added for 1 h after which the blot was thoroughly washed. Upon addition of the substrate (West Dura Extended Duration Chemiluminescent Substrate, Thermo Scientific) the signal was detected using a chemidoc XRS molecular imager (Bio-Rad) and spots individually quantified. To measure the intensity of reaction for each peptide, either a visual quantification was made on a scale from 0 (absent) to 5 (very strong) or a densitometric analysis of the signals was performed using Quantity One quantification analysis software (Bio-Rad). In the first array, because a high number or peptides were used (597), and since peptide arrays can be stripped using aqueous chaotropes (Abcam), the same membrane was used with three different serum samples. Nevertheless, to reduce non-specific background derived from stripping, in the 4 subsequent arrays, instead of testing the different samples in the same membrane after stripping, groups of peptides were spotted repeated times (6 in the second array, 9 in the third array, 10 in the fourth array, and 15 in the fifth array), membranes were cut into pieces, and each piece tested with a different serum.

### Statistical Analysis

A contingency table was created and a chi-squared (χ^2^) test was performed in the GRA7 peptide ELISA. A *p* < 0.01 was considered as significant.

## Results

### Peptide Arrays Identify Novel Antigenic and Strain-Specific Peptides

Five peptide array assays were performed in this study. The initial arrays aimed to identify polymorphic peptides that were antigenic (i.e., react well with sera from mice infected with at least one strain).

**Figure 1:**
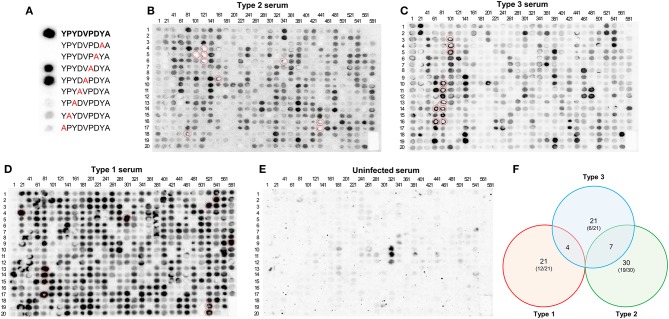
A *Toxoplasma* peptide array detects novel antigenic peptides. **(A)** HA-tag alanine-scan control. Reactivity of different Alanine-variants of the HA-tag epitope was measured with an HA-specific antibody. In bold, full HA amino acid sequence. In red, alanine substitutions. **(B)** A nitrocellulose membrane coated with 597 different peptides was blocked and incubated with serum from mice chronically infected with Pru (type 2) strain. After washing, the array was incubated with anti-mouse HRP antibody, washed, and incubated with luminescent substrate. Luminescent signal was detected using a CCD camera (shown as dark round spots or when signal was really strong and saturated as bright white spots). The membrane was subsequently stripped and then incubated again with **(C)** CEP (type 3), **(D)** RH (type 1), and **(E)** uninfected mouse serum, following the same procedure as described above. The peptide positions are indicated by numbers above each column (representing the number of the first peptide of the column) and on the left of each row (indicating the peptide position as a reference for each one of the columns). For instance, the dot on the intersection of the column named 81 and the row named 12 would be peptide number 92. Note the strong reactivity of several peptides (marked with red circles). For details on each peptide sequence readers are referred to Supplemental Table 2. **(F)** Venn diagram showing the number of specific and shared reactive (visual score ≥4) epitopes between type 1, 2, and 3 strains. Numbers in parenthesis indicate the proportion of specific peptides derived from novel proteins not previously used for serological assays.

The first array contained 597 peptides, including an alanine scan of the HA peptide as a positive control. Mouse sera from type 1 (RH), 2 (Pru), and 3 (CEP) infected mice and uninfected mouse serum as a negative control were used. As expected, there was strong reactivity against the intact HA peptide after incubation with an anti-HA monoclonal antibody, which was not affected when the amino acid was changed to an alanine at positions 5 or 6, but was almost completely abrogated when it was changed in the other positions (Figure 1A). From this control it was clear that polymorphisms at certain sites in a peptide do not affect serum reactivity, whereas mutations at other sites abrogate reactivity. To identify antigenic peptides, we pinpointed those that reacted strongly to one or several serum samples from the different types (Figures 1B–E). Eighty-three peptides from 26 different *Toxoplasma* proteins reacted strongly with at least one serum (score of at least 4, where 0 is absence of reaction and 5 the strongest reaction, see materials and methods) (Supplemental Table 2). While the majority of these 83 peptides were type-specific, 11 shared reactivity between types (4 reacted with both I and III whereas 7 reacted with both II and III sera) (Figure 1F). Some proteins, such as GRA3, GRA5-8, GRA15, ROP1, ROP18, SAG1, and SAG2A are known to be immunogenic and epitopes from these proteins were evaluated previously for their seroreactivity (Supplemental Table 1). In addition, peptides from novel antigenic proteins also reacted strongly to one or several serum samples, including GRA9, GRA31, ROP9, ROP10, ROP16, ROP19A, ROP20, ROP25, ROP26, ROP39, ROP47, TEEGR, Toxofilin, a histidine acid phosphatase superfamily protein (TGME49_308950), a putative oocyst wall protein (OWP, TGME49_222940), and one hypothetical protein (TGME49_268790) (Supplemental Table 2). Although our objective for this initial array was to identify immunogenic peptides, strain-specific peptides were also found. Forty-eight of the 83 antigenic peptides reacted to serum in a strain-specific manner with a reactivity score between strain types that differed by at least 3.5, however, only 15 of these polymorphic peptides reacted according to their strain type. In all, a total of 29 peptides reacted stronger with their type-specific serum, having a reactivity score >3.5, whereas their non-type reactivity score was equal or inferior to 2.5, indicating that the peptide was immunogenic across strains. Among these, type 1, 2, 3, 1/2, 1/3, and 2/3 specific peptides were identified (Supplemental Table 2). In addition, the uninfected serum sample was unreactive to all peptides except for three ROP16 peptides, which showed non-specific reactivity (Figure 1E).

**Figure 2:**
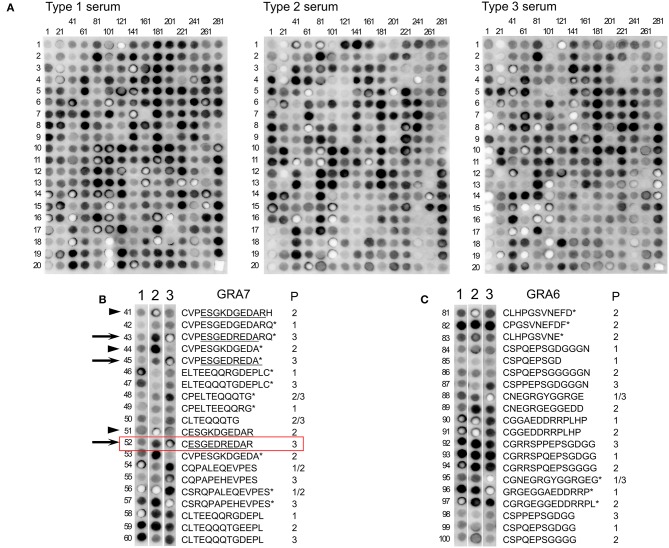
A second *Toxoplasma* peptide array detects many new strain-specific peptides. **(A)** Nitrocellulose membranes coated with 300 peptides was blocked and incubated with serum from mice chronically infected with RH (type 1, top left), ME49 (type 2, top middle), and VEG (type 3, top right) strains. After washing, the array was incubated with anti-mouse HRP antibody, washed, and incubated with luminescent substrate. Luminescent signal was detected using a CCD camera (shown as dark round spots or when signal was really strong and saturated as bright white spots). The peptide positions are indicated by numbers above each column (representing the number of the first peptide of the column) and on the left of each row (indicating the peptide position as a reference for each one of the columns). For details on each peptide sequence readers are referred to Supplemental Table 2. **(B)** Comparison of the third column of each membrane showing peptides 41–60 corresponding to different parts of GRA7 incubated with the different strain sera (indicated by numbers over each membrane strip). For each peptide the sequence is indicated and what strain(s) have that sequence. Type 3 peptides 43, 45, and 52 (arrows), and Type 2 peptides 41, 44, and 51 (arrowheads) are marked as examples to indicate the minimal antigenic peptide that confers a strong antigenicity (underlined). Peptide 52, which was further analyzed by ELISA (Figure 4), is marked with a box. **(C)** Comparison of the fifth column of each membrane showing peptides 81–100 corresponding to different parts of GRA6 incubated with the different strain sera (indicated by numbers over each membrane strip). For each peptide the sequence is indicated and what strain(s) have that sequence. P, peptide. An asterisk (*) indicate peptides already described by Kong et al. (2003). Readers are referred to Supplemental Table 2 for further details.

To optimize the assay, new peptides were selected to construct a second array with 300 spots, using type 1 (RH), 2 (ME49), and 3 (VEG) murine sera and 3 human serum samples from infected but healthy donors. We removed those peptides that showed a weak reaction in array 1 and redesigned previous peptides developed for serotyping in an effort to improve their reactivity by making them shorter or by altering left or right the register of the peptide in a stepwise fashion. A complete display of the array comparing reactivity with sera from mice infected with type 1, 2, and 3 strains is shown in Figure 2A and Supplemental File 1. Eighty peptides that reacted strongly (visual score ≥4) to at least one of the mouse samples were identified (Supplemental Table 2). Apart from the proteins described in array 1, peptides from ROP5, 8, 17, and 19A; 2 hypothetical proteins (TGME49_200360 and TGME49_308970), a reticulon protein (TGME49_226430), and a zinc finger (CCCH type) motif-containing protein (TGME49_242090) were identified. Moreover, 16 of these 80 peptides showed strong type-specific reactivity (at least 3.5 difference between types). Namely, peptides derived from GRA6, GRA7, SAG2A, ROP16, toxofilin, and TGME49_268790. In addition to confirming the validity of previous strain-typing peptides, new peptides were identified that accurately predicted the infecting strain (Figure 2A). These included peptides from ROP16 (pos. 160), ROP18 (pos. 27), ROP19A (pos. 10), ROP20 (pos. 276), SAG1 (pos. 125), and toxofilin (pos. 197, 200 and 203) (Figure 2A and Supplemental File 1).

Moreover, this array allowed us to define the optimum, minimal antigenic peptides for GRA6 and GRA7. For example, by analyzing the reactivity of the type 3 GRA7 peptides 43 and 45 from Kong et al. (2003) against a suite of new peptides within that antigenic region, we concluded that the minimal peptide 52, ESGEDREDAR was sufficient to bind type-specific antibodies with both specificity and sensitivity from type 3 infection serum (Table 2, Figure 2B, arrows). Indeed, peptide 52 possessed the same strong reactivity that was observed in peptides 43 (which contained two extra N-terminal amino acids and an extra C-terminal amino acid, VP and Q, respectively) or 45 (peptide 43 but lacking the two final amino acids RQ). Similarly, the type 2 GRA7 peptides 41, 44, and 51 revealed that VP was not needed for strong reactivity using type 2 sera, although in this case the final amino acids seemed to exert influence, as peptide 44 had reduced, but still high, reactivity against type 2 serum (Table 2, Figure 2B, arrowheads). Our high throughput array was also able to pinpoint relevant polymorphisms between types that appeared to be more important for strain specificity. For example, the GRA7 type 1/2 peptide 56 originally described by Kong et al. (2003) had a much stronger reaction with types 1 and 2 sera, whereas the type 3 version, peptide 57, had a much stronger reaction with type 3 serum (Figure 2B). Similarly, the GRA6 type 2 peptide number 81 had a strong reactivity with type 2 and the type 3 peptide number 87 with type 3 sera, as observed previously (Kong et al., 2003) (Figure 2C).

**Table 2:** Several versions of GRA6 and GRA7 peptides show the importance that specific amino acids have in serum reactivity to these peptides.

			**Visual score**		
**Peptide sequence**	**Type**	**Spot #**	**Type 1**	**Type 2**	**Type 3**
**GRA6**					
CLHPGSVNEFDF	2	80	3	4.5	3.5
CLHPGSVNEFD	2	81	2.5	4.5	2.5
CPGSVNEFDF	2	82	3.5	3.5	3.5
CLHPGSVNE	2	83	2	3.5	2
CLHPERVNVFDY	1/3	77	1	1	5
CLHPERVNVFD	1/3	79	2.5	2	5
CLHPERVNV	1/3	78	1.5	1	2.5
**GRA7**					
CVPESGKDGEDARH	2	41	1	4.5	2
CESGKDGEDAR	2	51	1	5	2
CVPESGKDGEDA	2	44	1	3.5	0.5
CVPESGEDREDARQ	3	43	1	3	5
CVPESGEDREDA	3	45	0.5	2	4.5
CESGEDREDAR	3	52	1	2	4.5

*GRA6 and GRA7 peptide sequences and strain type used in the second array are shown together with the visual score when tested with type 1, 2, and 3 mouse sera. Red letters in the peptide sequences mark amino acids that differ between strains. Red numbers in the visual scores mark the expected type serum to react for each peptide*.

Finally, a further analysis of this array was carried out by incubating the membrane with 3 different human samples from infected healthy patients (Supplemental Figure 1). Although the strain causing the infection was unknown, 40 peptides reacted strongly to one or more of the samples with a visual score ≥4. Among these, we found peptides derived from GRA3, GRA5, GRA6, GRA8, GRA31, ROP1, ROP8, ROP16, ROP39, ROP47, SAG2A, and Toxofilin (Supplemental Table 2 and Supplemental Figure 1). From these proteins, some peptides from GRA8, GRA31, ROP1, ROP47, and Toxofilin reacted strongly only with human, but not mouse, serum samples.

### GRA6 and GRA7 C-Terminal Peptides Show 13 Different Sequence Combinations

Because the GRA6 and GRA7 peptides showed promising results, we analyzed the amino acid sequences from the 64 strains available on ToxoDB for the C-terminal region (the last 11 and 13 aa, respectively) for these two proteins. This allowed us to determine the possible sequence combinations that give rise to different allele groups (Table 3). For the GRA6 C-terminal region, a total of 6 different 11-aa peptides were identified. The antigenicity of these 6 different possible GRA6 peptides has been previously reported to be different (Vaudaux et al., 2010). On the other hand, only three possibilities were observed for the GRA7 C-terminal 13-aa peptide. When both peptides were aligned together, a total of 13 unique combinations were detected for the 64 strains available in ToxoDB (Table 3). This clearly indicates that different strain genotypes belonging to different haplogroups can share the same amino acid sequence within these immunogenic epitopes. For instance, the type 2 strains ME49 and PRU are genotypically distinct from MAS, TgCatBr25, B41, and GAB5-2007-GAL-DOM6 [Haplogroups 4, 8, 12, and 14 respectively (Lorenzi et al., 2016)], but they possess the same GRA6 sequence, hence the atypical strains would be predicted to have type 2 reactivity for this peptide. Equally, the type 3 strains VEG and CEP are genotypically distinct from MAS, CAST, TgCatBr5 and TgCatBr44 (Haplogroups 4, 7, 8, and 10 respectively), but they possess the same GRA7 sequence, so the atypical strains would be predicted to have type 3 reactivity at this peptide. Accordingly, all available GRA6 and GRA7 sequence combinations, when analyzed together, identify 13 different combinations that should be able to separate type 1 from 2 from 3 from 12 clonal type infections (Table 3).

**Table 3:** Amino acid sequence alignment from GRA6 and GRA7 C-terminal region in the 64 strains available on ToxoDB shows the possible combinations that give rise to different allele groups.

**Strains (ToxoDB)**	**GRA6 sequence**	**No. strains (ToxoDB)**	**Alignment**
VEG, TgShUS28, TgRsCr1, TgDogCo17, TgCtCo5, TgCatPRC3, TgCkBr141, TgCkCr1, TgCkCr10, TgCatBr72, TgCatBr26, BOF, SOU, ROD, VAND, RH-JSR, RH-88, RH, M7741, GUY-2003-MEL, GT1, GAB5-2007-GAL-DOM1, GAB3-2007-GAL-DOM2, CAST, BRC_TgH_18021, BRC_TgH_18003_GUY-MAT, BRC_TgH_18002_GUY-KOE, BRC_TgH_18001_GUY-DOS, TgCatBr9, FOU	LHPERVNVFDY	30	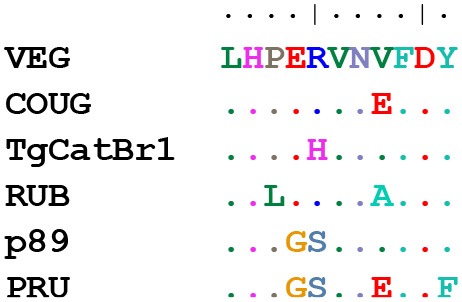
COUG, RAY, GUY-2004-JAG1, ARI, BRC_TgH_21016	LHPERVNEFDY	5	
TgCatBr1, TgCatBr3, TgCatBr44, BRC TgH 18009	LHPEHVNVFDY	4	
RUB	LHLERVNAFDY	1	
p89, GUY-2004-ABE, TgCatBr10, TgCatBr15, TgH26044, TgCkGy2, TgCatBr64, TgCatBr34, BRC_TgH_20005, CASTELLS, G662M. (GUY-TER, TOU-ALI and IppUrbB)	LHPGSVNVFDY	11	
PRU, TgCatBr18, TgCatBr25, TGME49, TgCATBr5, TgCat_PRC2, MAS, GAB5-2007-GAL-DOM6, GAB3-2007-GAL-DOM9, GAB2-2007-GAL-DOM2, GAB1-2007-GAL-DOM10, B41, B73	LHPGSVNEFDF	13	
**Strains (ToxoDB)**	**GRA7 sequence**	**No. strains (ToxoDB)**	**Alignment**
TGME49, PRU, SOU	VPESGKDGEDARQ	3	
TgShUS28, TgDogCo17, TgCkGy2, TgCkCr1, TgCkBr141, VAND, COUG, RAY, TgCat_PRC2, BOF, TgCatBr15, RH-JSR, RH-88, RH, GUY-2003-MEL, GT1, GAB5-2007-GAL-DOM6, GAB5-2007-GAL-DOM1, GAB3-2007-GAL-DOM9, GAB3-2007-GAL-DOM2, GAB2-2007-GAL-DOM2, GAB1-2007-GAL-DOM10, FOU, GUY-2004-ABE, BRC_TgH_18001_GUY-DOS, RUB, BRC_TgH_18021, BRC_TgH_18009, BRC_TgH_18002_GUY-KOE, BRC_TgH_18003_GUY-MAT, GUY-2004-JAG1, ARI, B41. (GUY-TER and TOU-ALI)	VPESGEDGEDARQ	33	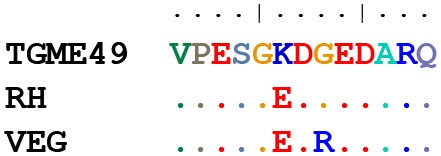
p89, VEG, TgRsCr1, TgCtCo5, TgCatPRC3, TgCatBr64, TgCatBr3, TgH26044, TgCatBr72, TgCatBr44, TgCatBr34, B73, TgCkCr10, ROD, M7741, G662M, CASTELLS, CAST, BRC_TgH_21016, MAS, TgCATBr5, TgCATBr9, TgCatBr1, TgCatBr10, TgCatBr18, TgCatBr25, TgCatBr26, BRC_TgH_20005. (IppUrbB)	VPESGEDREDARQ	28	
**Strains (ToxoDB)**	**GRA6///GRA7 sequence**	**No. strains (ToxoDB)**	**Alignment**
RH, RH-88, RH-JSR, GT1, FOU, BOF, BRC_TgH_18002_GUY-KOE, TgCkCr1, TgCkBr141, TgShUS28, TgDogCo17, BRC_TgH_18003_GUY-MAT, BRC_TgH_18021, BRC_TgH_18001_GUY-DOS, GAB3-2007-GAL-DOM2, GAB5-2007-GAL-DOM1, GUY-2003-MEL, VAND	LHPERVNVFDY///VPESGEDGEDARQ	18	
BRC_TgH_18009	LHPEHVNVFDY///VPESGEDGEDARQ	1	
ARI, RAY, COUG, GUY-2004-JAG1	LHPERVNEFDY///VPESGEDGEDARQ	4	
GUY-2004-ABE, TgCatBr15, TgCkGy2	LHPGSVNVFDY///VPESGEDGEDARQ	3	
RUB	LHLERVNAFDY///VPESGEDGEDARQ	1	
GAB1-2007-GAL-DOM10, GAB2-2007-GAL-DOM2, GAB3-2007-GAL-DOM9, GAB5-2007-GAL-DOM6, TgCat_PRC2, B41	LHPGSVNEFDF///VPESGEDGEDARQ	6	
BRC_TgH_21016	LHPERVNEFDY///VPESGEDREDARQ	1	
VEG, CAST, M7741, ROD, TgRsCr1, TgCATBr9, TgCatBr26, TgCatBr72, TgCkCr10, TgCtCo5, TgCatPRC3	LHPERVNVFDY///VPESGEDREDARQ	11	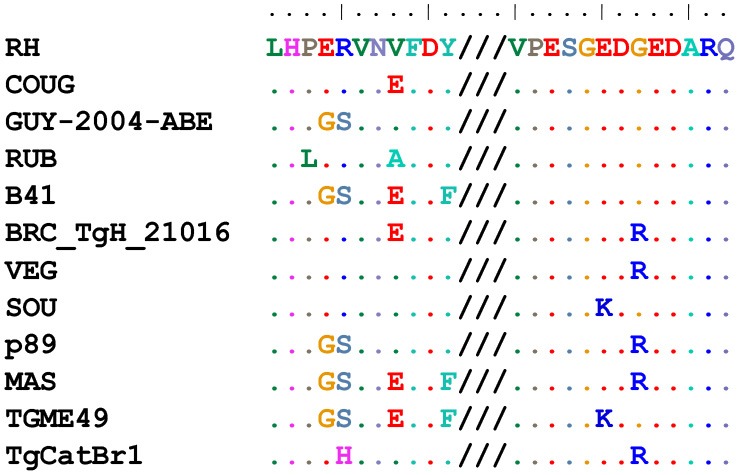
SOU	LHPERVNVFDY///VPESGKDGEDARQ	1	
p89, BRC_TgH_20005, CASTELLS, G662M, TgH26044, TgCatBr34,TgCatBr64, TgCatBr10	LHPGSVNVFDY///VPESGEDREDARQ	8	
MAS, B73, TgCatBr18, TgCatBr25, TgCATBr5	LHPGSVNEFDF///VPESGEDREDARQ	5	
ME49, Pru	LHPGSVNEFDF///VPESGKDGEDARQ	2	
TgCatBr1, TgCatBr3, TgCatBr44	LHPEHVNVFDY///VPESGEDREDARQ	3	

*The different possible sequences of GRA6 (above), GRA7 (middle) and a combination of both (bottom) are aligned and a representative strain from each group is given, where dots indicate identical amino acids. Red letters in the second column mark amino acids that differ between strains groups*.

Moreover, the arrays showed that some peptide regions in particular proteins are antigenic in some strains, but not for others. For example, type 1 mouse serum did not react to the type 1 GRA7 peptide CVPESGEDGEDARQ, whereas type 2 and 3 serum samples reacted strongly to the type 2 (CVPESGKDGEDARQ) and 3 (CVPESGEDREDARQ) versions of the peptide, respectively. By contrast, type 1 serum reacted strongly against a different GRA7 peptide (type 1 ELTEEQQRGDEPL), whereas the type 3 serum only reacted weakly against the equivalent type 3 version (ELTEQQQTGDEPL) (Figure 2B). Altogether, this suggests that the antibody response is focused on different regions of a protein in an epitope-dependent manner, which serves the diagnostic genotyping purposes.

### Optimization of Promising Peptides

For the third array, 100 peptides that reacted strongly and/or specifically in array 2 were repeated, some of them slightly modified (Supplemental Table 1), and tested using a panel of sera from mice infected with RH (type 1), FORT (type 2), WIL (type 2), and C56 (type 3), and rabbits infected with RH (type 1), ME49 (type 2), WIL (type 2), and VEG (type 3). A complete display of the third array comparing reactivity with sera from mice and rabbits is shown in Supplemental File 2. A strong reactivity (>40,000 integrated density) was observed for 32 and 27 peptides when incubated with mouse and rabbit sera, respectively. Among these, peptides from GRA3, GRA6, ROP1, ROP5, ROP16, ROP17, ROP18, ROP20, SAG2A, and TGME49_200360 reacted strongly in both mouse and rabbit serum samples. Moreover, some peptides were able to specifically recognize type 1 (ROP16 in rabbits), type 2 (GRA3, GRA5, GRA6, and GRA7 in mice; GRA3, SAG3, and ROP16 in rabbits) and type 3 (GRA5, GRA7, and ROP16 in mice) serum samples (Supplemental Table 2 and Supplemental File 2).

In the fourth array, we repeated some of the better peptides and redesigned others from the previous arrays. OT patients and healthy infected donor samples were used to test this array containing 120 peptide sequences (Supplemental File 3). Thirty-five peptides showed a strong reactivity (≥4 visual score), while 25 peptides reacted to at least 2 of the 10 samples with a visual score ≥3. Strong reactive peptides derived mainly from GRA (3, 5–9, 14–15) and ROP (5, 8, 16–18, 19A, 20, 26, 39–40) proteins, as well as SAG1, SAG3, one hypothetical protein (TGME49_268790), Toxofilin and a zinc finger (CCCH type) motif-containing protein (TGME49_242090). Among these, peptides on position 42 (GRA15), 71 (ROP8), and 93 (TGME49_242090) reacted strongly to several of the samples tested (Supplemental Table 2 and Supplemental File 3). In addition, it is worth mentioning that two of the samples, Patient 5 and Donor 2, despite possessing similar titers as that found for all sera tested by ELISA against whole antigen, did not react to any of the 120 peptides from this array with a visual score of 3 or more. Only 2 peptides were able to elicit a reaction with a visual score of 2.5 in Patient 5: ROP19A (position 105) and ROP8 (position 74). By contrast, Donor 1 showed a high reactivity (visual score of 3 or more) with 44 of the 120 peptides (Supplemental Table 2 and Supplemental File 3). All sera samples were titered into the same range (1/1200-1/4800) using whole *Toxoplasma* antigen to avoid significant differences in the antibody levels. Hence, differences in reactivity could not be simply attributed to each sample's titer.

**Figure 3:**
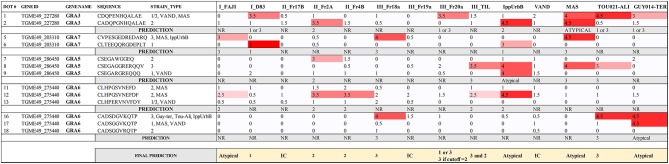
Serotyping results for human serum samples using a combination of different GRA3, GRA5, GRA6, and GRA7 peptides. The strain that caused the infection for each sample is shown and the visual score is indicated for each peptide, being 0 absence of reaction, and 5 the strongest reaction. A prediction for each sample is made based on the results obtained for each peptide. IC, inconclusive; NR, non-reactive.

Finally, the fifth array was made with the same or slightly different sequences from the 40 best peptides in the previous arrays, and a total of 14 serum samples from which the strain causing the infection is known, as well as a few unknown human serum samples were used. A complete display of the fifth array comparing these samples is shown in Supplemental File 4. From the 40 tested peptides, 18 showed a visual score of ≥4. These peptides were derived from GRA (3, 5–8, 15) and ROP (8, 19A, 20, 39) proteins. In addition, 13 peptides reacted with at least 3 of the samples with a visual score of 3.5 or higher, including, apart from the above mentioned, a ROP26 derived peptide. Moreover, 2 of the 15 samples (VAND and III-Fr20a) did not react to any of the peptides with a visual score higher than 3.5, and only a TEEGR (position 25) and a GRA3 (position 1) peptide reacted with a visual score of 3.5 against VAND and IIIFr20a, respectively (Supplemental Table 2). As described for array 4, titers were similar among all samples used in this array and different dilutions were made to ensure similar levels of antibodies were present in all the samples. Unfortunately, the last two arrays using human serum did not detect strain-specific peptides able to accurately predict the genotype of the human infection for all of the human samples used. Nevertheless, when a visual score of 2.5 was considered as a cut-off, a number of peptides derived from GRA3, 5, 6, and 7 predicted the strain in several serum samples (Figure 3). From the 14 serum samples, 8 were correctly predicted, 3 did not react to any of the selected peptide above the cut-off limit and the remaining 3 had mixed reaction that rendered the prediction inconclusive. Moreover, and in contrast to archetypal strains, IppUrbB, MAS and GUY-TER serum samples reacted strongly to different versions of the same peptide. IppUrbB sample was a case of reinfection in a pregnant woman leading to congenital infection (Elbez-Rubinstein et al., 2009) that was later identified as haplogroup 16 by microsatellite markers and whole genome sequencing (Lorenzi et al., 2016). Hence there was a mixture of reactions to several peptides.

### A Novel GRA7 Peptide Specifically Detects Type 3 Infections

**Figure 4:**
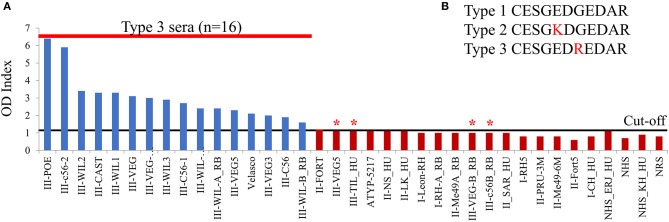
A novel GRA7 peptide identifies type 3 strain infections with high specificity and sensitivity. Our peptide arrays identified a GRA7 peptide that worked well in distinguishing infections with type 3 strains and was therefore synthesized, coupled to KLH and loaded into an ELISA plate. Sera from mice, rabbits (RB), and humans (HU) infected with types 1 (I), 2 (II), or 3 (III) strains was added and **(A)** binding was detected using an HRP-conjugated secondary antibody using ELISA assay according to Kong et al. (2003). The peptide has high specificity with no false positives detected, however four type 3 sera failed to react (indicated by *). OD, Optical Density. Cut-off value was 1.4, above which samples were considered positive. **(B)** GRA7 peptide sequences for the three archetypal strains.

Overall, the arrays carried out trying different versions of the same peptides with different serum samples allowed us to identify a large number of antigenic and strain-specific peptides using the peptide array approach. These were derived from several proteins, many of which are described here for the first time to have diagnostic potential. However, arrays are not always readily available, they are expensive and are not easily transferable to other laboratories. Hence, as a proof-of-concept, we tested the ELISA protocol originally used for serotyping type 2 from non-type 2 infections (Kong et al., 2003). The array identified a delimited version of a type 3-specific peptide from GRA7 (ESGEDREDAR) that could distinguish type 3 from type 1 and 2 infections (Figure 2B, position 52). To confirm this prediction, the peptide was tested against a broad panel of type-specific serum samples from mice, rabbit, and human from which the infecting strain was known. To this end, a cysteine residue was added to the C terminus so that it could be coupled to the carrier protein keyhole limpet hemocyanin (KLH) and it was synthesized as a soluble peptide. The ELISA results show that the delimited GRA7 peptide was able to accurately detect type 3 infections in mouse, rabbit and human samples (χ^2^ 20.29, *p* < 0.0001) (Figure 4) with high specificity, confirming the results observed in the second peptide array (Figure 2B). Indeed, 16 type 3 serum samples reacted to this peptide, with only 2 rabbit, 1 mouse and 1 human type 3 samples falling below the cut-off limit. No type 1 or 2 serum samples from any species reacted to this peptide indicating that it was both highly specific, with no false positives detected, and 80% sensitive, with only 4 false negatives. Furthermore, 3 out of 4 false negatives were from infections with the VEG strain, which could suggest a specific non-reactivity for this allelic epitope upon infection with this strain. Additionally, CAST, which is not a Type III strain, had a type 3 sequence at the GRA7 peptide epitope (Table 3) indicating that antibodies were not dependent on the allele present at GRA7, but rather on the epitope sequence, which is why CAST serotyped as type 3 at GRA7.

## Discussion

*Toxoplasma gondii* is known to cause a wide spectrum of clinical presentations in animals and humans, ranging from asymptomatic to severe, even lethal, disease. Besides the host immune response, it is known that strain type is one of the key-factors responsible for the clinical appearance of toxoplasmosis (Boothroyd and Grigg, 2002; Kong et al., 2003). To develop a rapid, sensitive and non-invasive method of identifying strain type, serotyping has been shown to provide a promising alternative to the not always possible, difficult and often risky biopsy-based DNA methods (Kong et al., 2003; Vaudaux et al., 2010). Indeed, several studies have employed synthetic peptides or recombinant polypeptides from polymorphic regions to serologically predict the clonal type of *T. gondii* responsible for the infection, showing that in hosts such as cats, mice, chickens, turkeys, pigs, sheep, or humans it is possible to reliably distinguish type 2 from non-type 2 infections (Kong et al., 2003; Peyron et al., 2006; Morisset et al., 2008; Sousa et al., 2009, 2010; Xiao et al., 2009; Vaudaux et al., 2010; Maksimov et al., 2012a,b, 2018; McLeod et al., 2012; Hutson et al., 2015). In addition, some studies attempted to develop peptides able to differentiate type 1 vs. 3 and type 2 vs. 3 infections with partial success (Xiao et al., 2009; Maksimov et al., 2012a, 2018). However, to date, a clear serotyping distinction between type 1, 2 and 3 strains, as well as those from non-archetypal strains, has not been possible.

In the present study a total of 950 peptides from 62 different polymorphic or differentially expressed *Toxoplasma* proteins were analyzed. For this, a large-scale peptide array assay was used to test different peptides with mice, rabbit and human sera, which allowed us to identify the most antigenic peptides. Some of these, such as GRA1, 3, 5–8, and 15; ROP1, 5, 8, 9, and 18; and SAG1, 2A, 3, have been already reported to be highly antigenic and proved useful in different *Toxoplasma* serodiagnostic techniques (e.g., Beghetto et al., 2003; Kong et al., 2003; Grzybowski et al., 2015). However, we describe here for the first time the potential use of a large number of immunogenic peptides derived from proteins that have never been used for serodiagnosis purposes before, such as GRA9, GRA31, ROP10, ROP16, ROP20, ROP25, ROP26, ROP38, ROP39, or toxofilin, among others (Supplemental Tables 1, 2). Unsurprisingly, peptides derived from GRA5, 6 and 7 were the most reactive, which correlates with the high potential of dense granule proteins as diagnostic antigens shown in previous works (e.g., Maksimov et al., 2012a). Moreover, our results revealed a number of peptides with the ability to discriminate between mice infected with different type 1, 2 and 3 strains. As mentioned above, peptides able to distinguish type 2 vs. non-type 2 (Kong et al., 2003), type 1 vs. type 3 (Xiao et al., 2009), and type 2 vs. type 3 (Maksimov et al., 2012a) infections were previously described. Herein, to the best of our knowledge, we describe for the first time peptide combinations with the potential of differentiating type 1 vs. type 2 (e. g. GRA7, ROP17, and ROP18), as well as novel peptides that also successfully discriminate type 1 vs. 3 (e.g., SAG2A, ROP20, and Toxofilin) and 3 vs. non-3 (e.g., GRA7 and ROP19A). These new peptides warrant further investigation using a broader panel of serum samples from different animals and conditions to test their ability to efficiently discriminate between infections caused by different *Toxoplasma* strains. Nevertheless, the final objective of *Toxoplasma* serotyping is arguably aimed to assist in human toxoplasmosis, and as such we included human samples in our arrays. However, because the human sera were available in limited quantities, they were not used for the initial screening of large numbers of peptides. Therefore, the usage of genotyped human patients was restricted to the last arrays in which the selection of peptides was narrowed down. A large number of peptides were very immunogenic in humans, and most of these were also antigenic in mice. Unfortunately, none of the peptides tested in the final arrays were able to accurately discriminate all patients infected with different strains. Hence, a further investigation with a broader panel of characterized samples from patients is needed. Nonetheless, when a panel of GRA3, GRA5, GRA6, and GRA7 peptides were selected using a lower cut-off, several human serum samples were correctly predicted. As reported by others (e.g., Kong et al., 2003; Sousa et al., 2008) patients infected with non-archetypal strains such as MAS, GUY-TER, and IppUrbB often possess strong reactions with peptides harboring type 1, 2, and/or 3 lineage sequences. However, upon closer examination, MAS, which has a type 4 genotype, possesses type 2 peptide epitopes at GRA6 and GRA7, which is why it produces a type 2 serotype. In comparison, serum from IppUrbB, a pregnant woman that became re-infected during gestation leading to congenital transmission (Elbez-Rubinstein et al., 2009), possessed dual reactivity because the second infecting strain was later identified to belong to haplogroup 16 (Lorenzi et al., 2016), which explained her mixed reactivity serotyping pattern. It is worth mentioning that the serotyping methodology showed a high degree of individual variability for some peptides depending on the strain initiating infection. In fact, several peptides that reacted strongly to serum from mice infected with the corresponding peptide epitope, also reacted with sera from animals infected with strains that did not possess the cognate peptide sequence. By contrast, other peptides were only weakly recognized by a limited number of sera from animals infected with strains that had the respective type-specific peptide sequence. Because of this mixture of cross-reactivity and/or low sensitivity, just as PCR-RFLP for a single locus cannot determine strain type, serotyping undoubtedly requires a large number of polymorphic peptides from different antigens to be analyzed as a whole, instead of relying on individual peptides, to provide a serological profile or signature that defines a strain type (Sousa et al., 2008; Vaudaux et al., 2010; Maksimov et al., 2012a). Likewise, we observed a small proportion of human sera that did not react to any of the peptides tested, whereas other sera reacted strongly to a high number of peptides. Since the titer of the samples were similar and dilutions were performed accordingly, it is unlikely that this was due to differences in the level of antibodies. The great individual variability in human samples detected here is in accordance with previous reports (Kong et al., 2003; Sousa et al., 2009; Maksimov et al., 2012a). This may be due to the different conditions of individual patients, such as immunocompetence status, stage of the disease (chronic vs. acute), mixed infections, different sources of infection (oocysts vs. tissue cysts), HLA alleles, or age that could exert a strong influence in the presence or absence of specific antibodies that react to very few or a high number of epitopes in the parasite antigens. Therefore, this reinforces the notion that the use of single or few peptides for serotyping may lead to mistyping and hence a large pool of polymorphic peptides from different antigens should be used.

In addition to the low type-specificity of particular peptides, previous work has shown that strongly immunoreactive peptides may generate sufficient antibodies to non-polymorphic amino acids within a polymorphic peptide, i.e., reactivity does not necessarily predict strain serotype (Vaudaux et al., 2010). A similar effect was evident herein, some peptides for which the sequence was identical in types 1 and 2, 1 and 3, or 2 and 3 showed a much stronger reaction to one of them, but not both. This may be accounted for by the presence of other polymorphic immunogenic epitopes in the protein of certain strains that are dominant and prevent other epitopes from reacting. This may render the polymorphisms irrelevant in terms of strain reactivity. For example, if region A is dominant in one strain, it is unlikely that the reactivity against peptides on region B is high, regardless of the polymorphic match with that particular strain. On the contrary, it may be possible that the reactivity of other strains, even when they do not correspond to that particular peptide type, are stronger because region B is immunodominant in those strains. Although it may seem counterproductive, this fact may turn advantageous, as it can be used to identify certain strain types by their distinctive behavior to specific peptides. This fact may be particularly relevant in detecting non-archetypal strains: even if a particular isolate shares the sequence with type 1, 2, or 3 strains, the reactivity may not be the same. A clear example of this was the reactivity found in the human serum GUY-TER and TOU-ALI. Despite sharing the same amino acid sequence in two GRA6 and GRA7 peptides, the reactivity to these peptides was different. This fact can be used as a marker for the distinction between these two non-archetypal strains.

Similarly, a different amino acid sequence may not preclude the samples from reacting against the archetypal version of a specific peptide. Hence, by characterizing the unique reactivity of a particular non-archetypal strain with peptides derived from the type 1, 2, and 3 lineages, irrespective of the amino acid sequence similarity, a characteristic signature of that strain can be determined, which in turn can be used in the future for its identification. Regardless, when the amino acid sequences of GRA6 and GRA7 C-terminal peptides were compared between the 64 strains available at ToxoDB, only 6 and 3 different possibilities were, respectively, detected, which increased to 13 combinations when both peptides were compared together. These different peptides have been previously shown to be antigenically different (Vaudaux et al., 2010). As a consequence, strains belonging to different haplogroups can have similar reactivity to a peptide (e.g., type 2 has the same GRA6 C-terminal sequence as MAS), but similarly all strains allocated in the same haplogroup may not possess the same sequence. This can be explained by the influence of recombination on the global population structure of *T. gondii* (Lorenzi et al., 2016). Indeed, many strains that are currently classified as belonging to the same haplotype are genetically different, especially if they do not belong to one of the clonal lineages. This further supports the idea that it is the epitope, rather than the genetic type, that determines the specificity of the peptide (Vaudaux et al., 2010). As a matter of fact, atypical strains possessing different epitopes at GRA6 and GRA7 generally induce antibodies that either cross-react with non-polymorphic antigenic regions or do not react.

On the other hand, our results highlight the importance that single amino acid substitutions have on the peptide recognition. The best example of this is the F/Y amino acid in the C-terminal portion of GRA6. As pointed out previously, the deletion of this amino acid eliminated all false positive results (Kong et al., 2003). In the present work we further investigated the importance of some amino acids compared to others by designing different versions that were shorter or longer and that varied at different levels in amino acid composition between strains. Our results indicate and confirm that in the GRA6 C-terminal, the GS/ER polymorphisms are not important, while the EFDF/VFDY portion is much more relevant. Similarly, the most important amino acid in a group of GRA7 peptides seems to be the K in position 229. This changes to E in all the other strains that remain unreactive. These results clearly indicate that the polymorphic site and length of peptide play a significant role in serotyping, in accordance to previous studies (Kong et al., 2003). When comparing the average length of our set of peptides with other studies (Sousa et al., 2008; Xiao et al., 2009; Maksimov et al., 2013), the shorter peptides may improve the specificity, but affect sensitivity. However, in longer peptides, the large number of conserved or invariant amino acids that do not predict strain type often increase the cross-reactivity and thus the false positive results (Sousa et al., 2009). In addition, the alignment of the 64 strains available in ToxoDB revealed a number of possible combinations of amino acid sequences for different peptides. When we analyzed the GRA6 and GRA7 C-terminal peptides, a total of 6 and 3 different combinations of alleles were, respectively, identified. In theory, the same number of peptides could be designed to identify the respective strains. However, as mentioned above, certain polymorphisms seem to have a much stronger impact on reactivity than others. Hence, in practice fewer than the number of possible peptides will discriminate between strain types. Future experiments should be conducted to determine whether peptides from the different alleles are strain specific. This could prove important in identifying atypical strains by using archetypal-based peptides, even when the sequence is not exactly the same.

Finally, although for logistic reasons we did not further investigate all the peptides derived from proteins that were antigenic in the arrays, these novel proteins possess potential diagnostic properties that could be further tested in future experiments. Notwithstanding, as a proof of concept, an ELISA was performed with one of the most promising peptides identified in our arrays: ESGEDREDAR. This novel peptide was derived from GRA7 and shown to effectively discriminate type 3 infection in mice, rabbit and human serum samples with a high specificity and sensitivity. By following this example, more promising peptides could be tested with a larger collection of gold standard samples by using an easier, more accessible to all laboratories and affordable test such as ELISA. Nevertheless, the need for a large pool of polymorphic peptides from different loci is of paramount importance in order to define all the different profiles from each strain and to compensate for the high individual variability shown by both individual peptides and samples. By increasing the number of peptides that can be recognized by an individual serum and allow it to be classified, the strain a person is infected with might be unambiguously defined, as happens with current RFLP methods. This, in turn, will allow large studies to be conducted that can correlate the genotype of the infecting strains with disease outcome to better understand the molecular epidemiology of *Toxoplasma*.

## Data Availability Statement

All datasets generated for this study are included in the article/Supplementary Material.

## Ethics Statement

Serum samples from infected patients genotyped either in France (Center national de reference-Toxoplasmose Limoges France) (Sousa et al., 2008, 2009) or in the United States (de-la-Torre et al., 2013; Shobab et al., 2013) were used, as well as samples from healthy infected individuals and OT patients from Brazil but living in USA. Human samples were used according to the Committee on the Use of Humans as Experimental Subjects (COUHES) application No. 0808002869. The patients/participants provided their written informed consent to participate in this study. All mouse work was performed in accordance with the recommendations in the Guide to the Care and Use of Laboratory Animals (104) of the National Institutes of Health. The MIT Committee on Animal Care (assurance no. A-3125-01) and the Animal Care and Use Committee of the Intramural Research Program of NIAID (Animal Study Protocol LPD22E) approved all protocols, and all efforts were made to minimize unnecessary distress to the animals. Written informed consent was obtained from the individual(s) for the publication of any potentially identifiable images or data included in this article.

## Author Contributions

JS conceived the study and acquired funding. CC, LY, AC, MD, and MG provided the samples and carried out titration assays. CC conducted the array experiments. AC and MG performed the ELISA assay and analyzed the data. DA-S and JS analyzed the array data, edited the figures and tables and wrote the manuscript. DA-S, MG, and JS revised and edited the manuscript with input comments from CC, LY, and MD. All authors read and agreed on the final version of the manuscript.

### Conflict of Interest

The authors declare that the research was conducted in the absence of any commercial or financial relationships that could be construed as a potential conflict of interest.

## Supplementary Material

Supplemental Figure 1 Second peptide array incubated with human serum samples. A nitrocellulose membrane coated with 300 different peptides was blocked and incubated with serum from 3 different humans infected with *Toxoplasma* but the strain that caused the infection is unknown. After washing, the array was incubated with anti-human HRP antibody, washed, and incubated with luminescent substrate. Luminescent signal was detected using a CCD camera (shown as dark round spots or when signal was really strong and saturated as bright white spots). The peptide positions are indicated by numbers on the left of each row (representing the number of the first peptide of the row) and above each column (indicating the peptide position as a reference for each one of the rows). For instance, the dot on the intersection of the column named 14 and the row named 61 would be peptide number 74.

Supplemental Table 1 List of proteins and peptides used in the present study.

Supplemental Table 2 Quantification of peptide arrays.

Supplemental File 1 Strips from array 2 comparing individual peptides for each serum sample from infected mice. Strips from each array incubated with the different samples were taken and put together as a comparison. Peptide numbers are indicated above each group of strips. Strain types are indicated on the left side of each strip: RH (type 1), Pru (type 2), and VEG (type 3).

Supplemental File 2 Strips from array 3 comparing individual peptides for each serum sample from infected mice and rabbits. Strips from each array incubated with the different samples were taken and put together as a comparison. Peptide numbers are indicated above each group of strips. Strain names are indicated on the left side of each strip: RH (type 1), FORT (type 2), WIL (type 2), and C56 (type 3) in mice and RH (type 1), ME49 (type 2), WIL (type 2), and VEG (type 3) in rabbits. **(A,B)** indicates two different samples from the same group of animals.

Supplemental File 3 Strips from array 4 comparing individual peptides for each serum sample from human patients. Strips from each array incubated with the different samples were taken and put together as a comparison. Peptide numbers are indicated above each group of strips. Patient identifications are indicated on the left side of each strip. Pt and Dn stand for Patient and Donor, respectively.

Supplemental File 4 Strips from array 5 comparing individual peptides for each serum sample from human patients. Strips from each array incubated with the different samples were taken and put together as a comparison. Peptide numbers are indicated above each group of strips. Patient identifications are indicated on the left side of each strip.
