# Supplementary figures and images for: Serotyping of Toxoplasma gondii Infection Using Peptide Membrane Arrays
Source: Front Cell Infect Microbiol. 2019 Nov 29;9:408. doi: 10.3389/fcimb.2019.00408 (PMC6895565; doi:10.3389/fcimb.2019.00408)

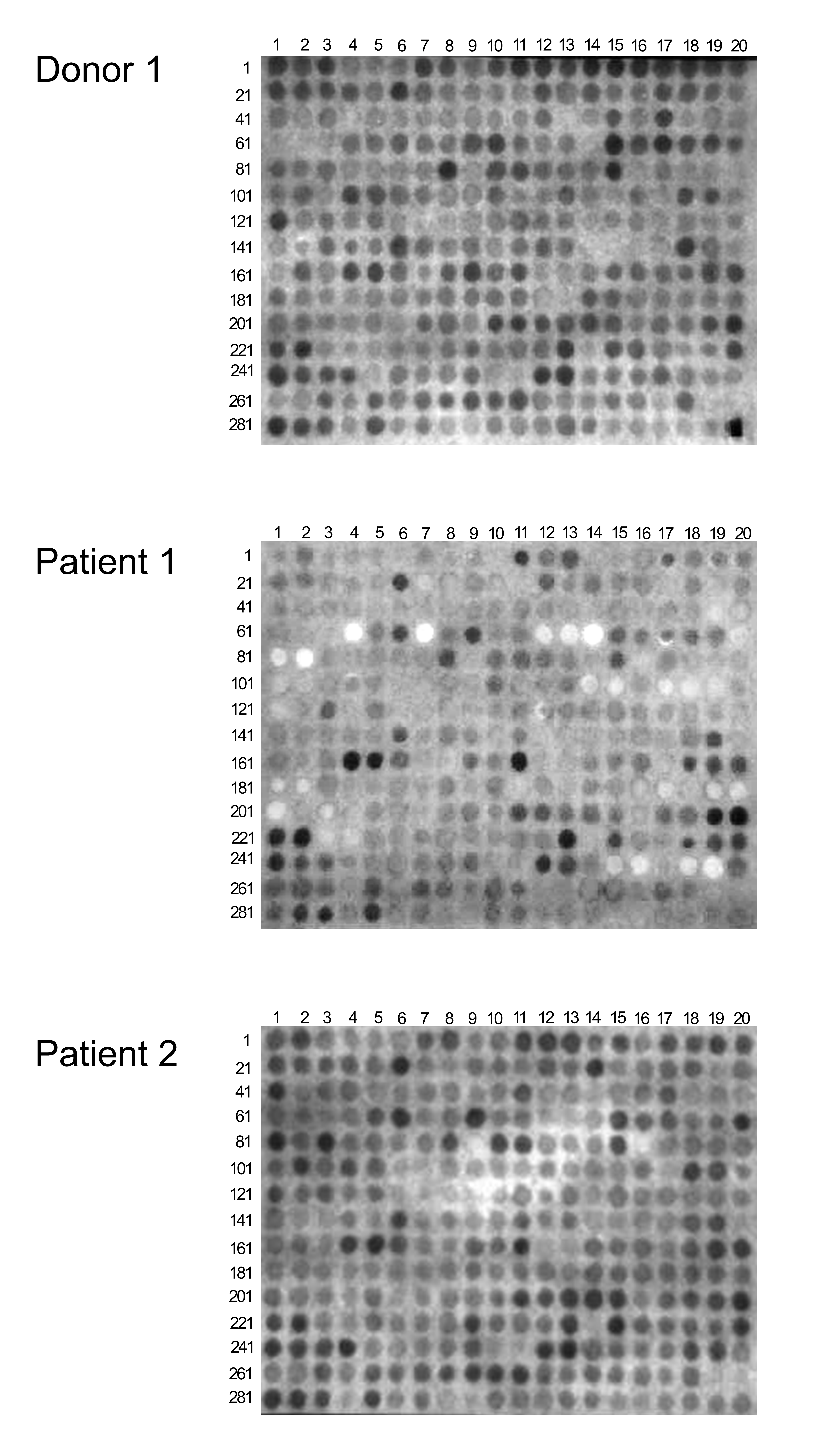

Supplement: Supplemental Figure 1 — Second peptide array incubated with human serum samples. A nitrocellulose membrane coated with 300 different peptides was blocked and incubated with serum from 3 different humans infected with Toxoplasma but the strain that caused the infection is unknown. After washing, the array was incubated with anti-human HRP antibody, washed, and incubated with luminescent substrate. Luminescent signal was detected using a CCD camera (shown as dark round spots or when signal was really strong and saturated as bright white spots). The peptide positions are indicated by numbers on the left of each row (representing the number of the first peptide of the row) and above each column (indicating the peptide position as a reference for each one of the rows). For instance, the dot on the intersection of the column named 14 and the row named 61 would be peptide number 74. [file Image_1.TIF]
