# Supplementary material for: Serotyping of Toxoplasma gondii Infection Using Peptide Membrane Arrays
Source: Front Cell Infect Microbiol. 2019 Nov 29;9:408. doi: 10.3389/fcimb.2019.00408 (PMC6895565; doi:10.3389/fcimb.2019.00408)
Supplement: Supplemental File 1 — Strips from array 2 comparing individual peptides for each serum sample from infected mice. Strips from each array incubated with the different samples were taken and put together as a comparison. Peptide numbers are indicated above each group of strips. Strain types are indicated on the left side of each strip: RH (type 1), Pru (type 2), and VEG (type 3). [file Presentation_1.pptx]

## Slide 1
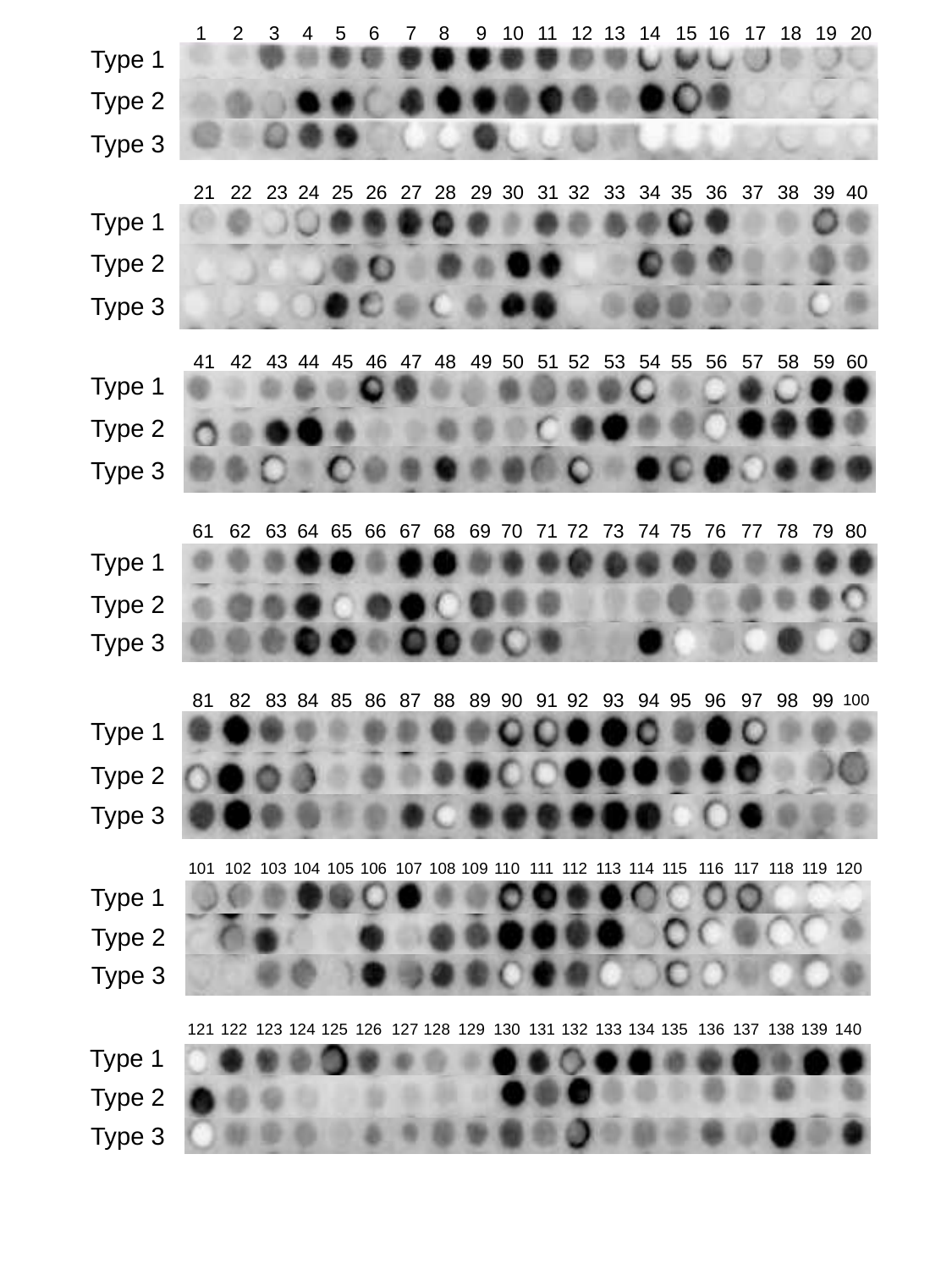

1
2
3
4
5
6
7
8
9
10
11
12
13
14
15
16
17
18
19
20
Type 1
Type 2
Type 3
21
22
23
24
25
26
27
28
29
30
31
32
33
34
35
36
37
38
39
40
Type 1
Type 2
Type 3
41
42
43
44
45
46
47
48
49
50
51
52
53
54
55
56
57
58
59
60
Type 1
Type 2
Type 3
61
62
63
64
65
66
67
68
69
70
71
72
73
74
75
76
77
78
79
80
Type 1
Type 2
Type 3
81
82
83
84
85
86
87
88
89
90
91
92
93
94
95
96
97
98
99
100
Type 1
Type 2
Type 3
101
102
103
104
105
106
107
108
109
110
111
112
113
114
115
116
117
118
119
120
Type 1
Type 2
Type 3
121
122
123
124
125
126
127
128
129
130
131
132
133
134
135
136
137
138
139
140
Type 1
Type 2
Type 3

## Slide 2
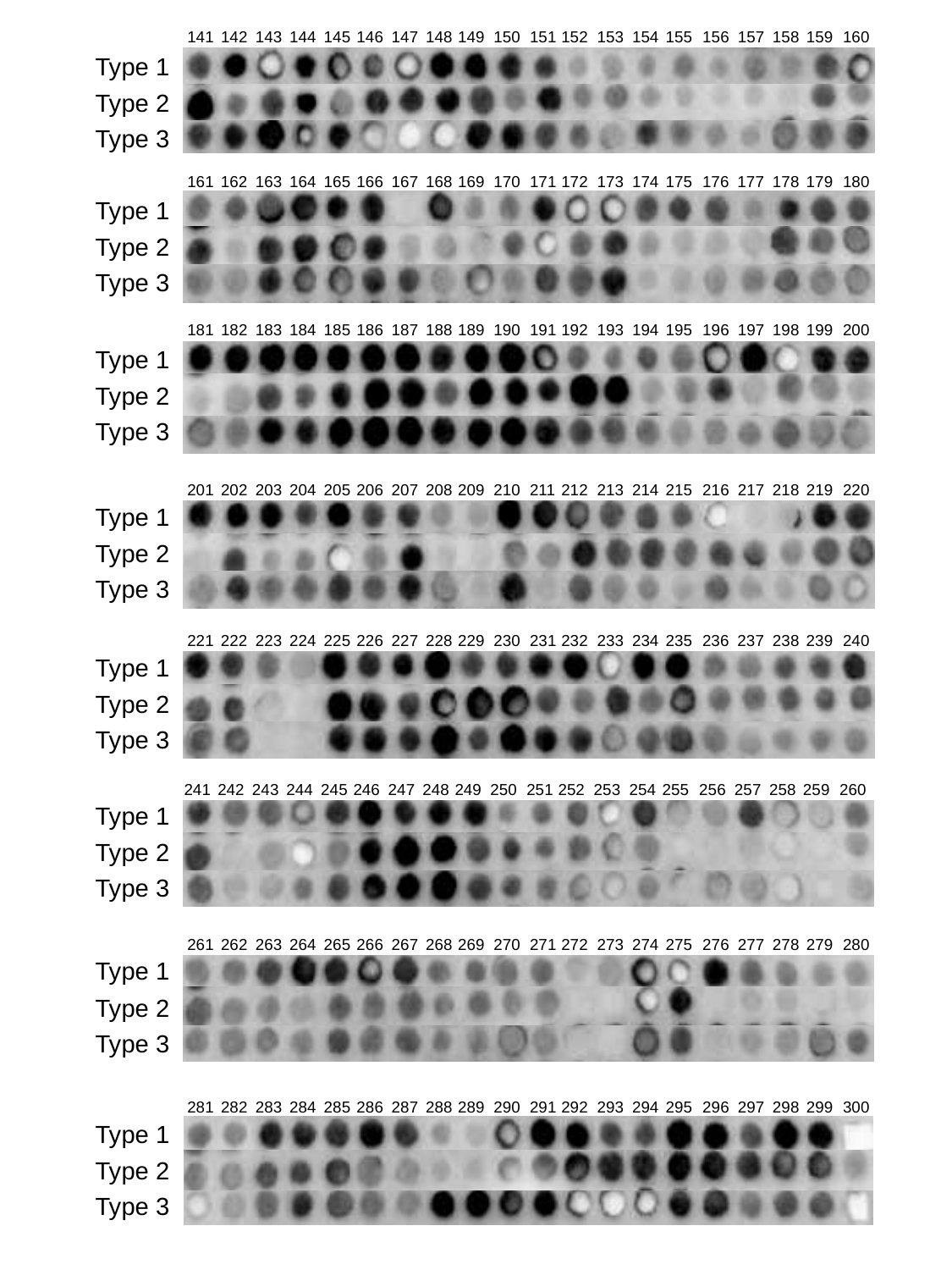

141
142
143
144
145
146
147
148
149
150
151
152
153
154
155
156
157
158
159
160
Type 1
Type 2
Type 3
161
162
163
164
165
166
167
168
169
170
171
172
173
174
175
176
177
178
179
180
Type 1
Type 2
Type 3
181
182
183
184
185
186
187
188
189
190
191
192
193
194
195
196
197
198
199
200
Type 1
Type 2
Type 3
201
202
203
204
205
206
207
208
209
210
211
212
213
214
215
216
217
218
219
220
Type 1
Type 2
Type 3
221
222
223
224
225
226
227
228
229
230
231
232
233
234
235
236
237
238
239
240
Type 1
Type 2
Type 3
241
242
243
244
245
246
247
248
249
250
251
252
253
254
255
256
257
258
259
260
Type 1
Type 2
Type 3
261
262
263
264
265
266
267
268
269
270
271
272
273
274
275
276
277
278
279
280
Type 1
Type 2
Type 3
281
282
283
284
285
286
287
288
289
290
291
292
293
294
295
296
297
298
299
300
Type 1
Type 2
Type 3
