# Supplementary material for: Serotyping of Toxoplasma gondii Infection Using Peptide Membrane Arrays
Source: Front Cell Infect Microbiol. 2019 Nov 29;9:408. doi: 10.3389/fcimb.2019.00408 (PMC6895565; doi:10.3389/fcimb.2019.00408)
Supplement: Supplemental File 2 — Strips from array 3 comparing individual peptides for each serum sample from infected mice and rabbits. Strips from each array incubated with the different samples were taken and put together as a comparison. Peptide numbers are indicated above each group of strips. Strain names are indicated on the left side of each strip: RH (type 1), FORT (type 2), WIL (type 2), and C56 (type 3) in mice and RH (type 1), ME49 (type 2), WIL (type 2), and VEG (type 3) in rabbits. (A,B) indicates two different samples from the same group of animals. [file Presentation_2.PPTX]

## Slide 1
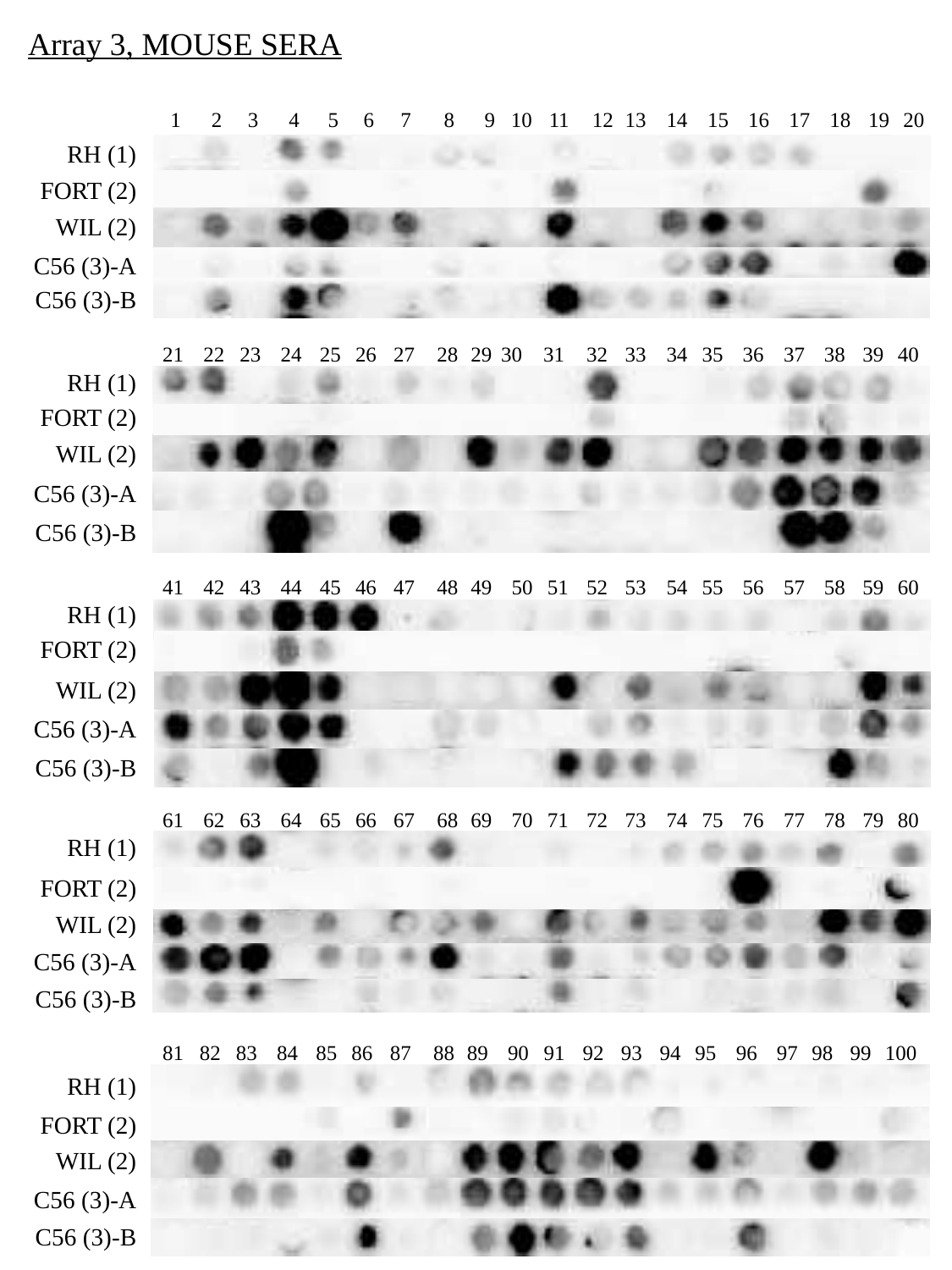

Array 3, MOUSE SERA
1
2
3
4
5
6
7
8
9
10
11
12
13
14
15
16
17
18
19
20
RH (1)
FORT (2)
WIL (2)
C56 (3)-A
C56 (3)-B
21
22
23
24
25
26
27
28
29
30
31
32
33
34
35
36
37
38
39
40
RH (1)
FORT (2)
WIL (2)
C56 (3)-A
C56 (3)-B
41
42
43
44
45
46
47
48
49
50
51
52
53
54
55
56
57
58
59
60
RH (1)
FORT (2)
WIL (2)
C56 (3)-A
C56 (3)-B
61
62
63
64
65
66
67
68
69
70
71
72
73
74
75
76
77
78
79
80
RH (1)
FORT (2)
WIL (2)
C56 (3)-A
C56 (3)-B
81
82
83
84
85
86
87
88
89
90
91
92
93
94
95
96
97
98
99
100
RH (1)
FORT (2)
WIL (2)
C56 (3)-A
C56 (3)-B

## Slide 2
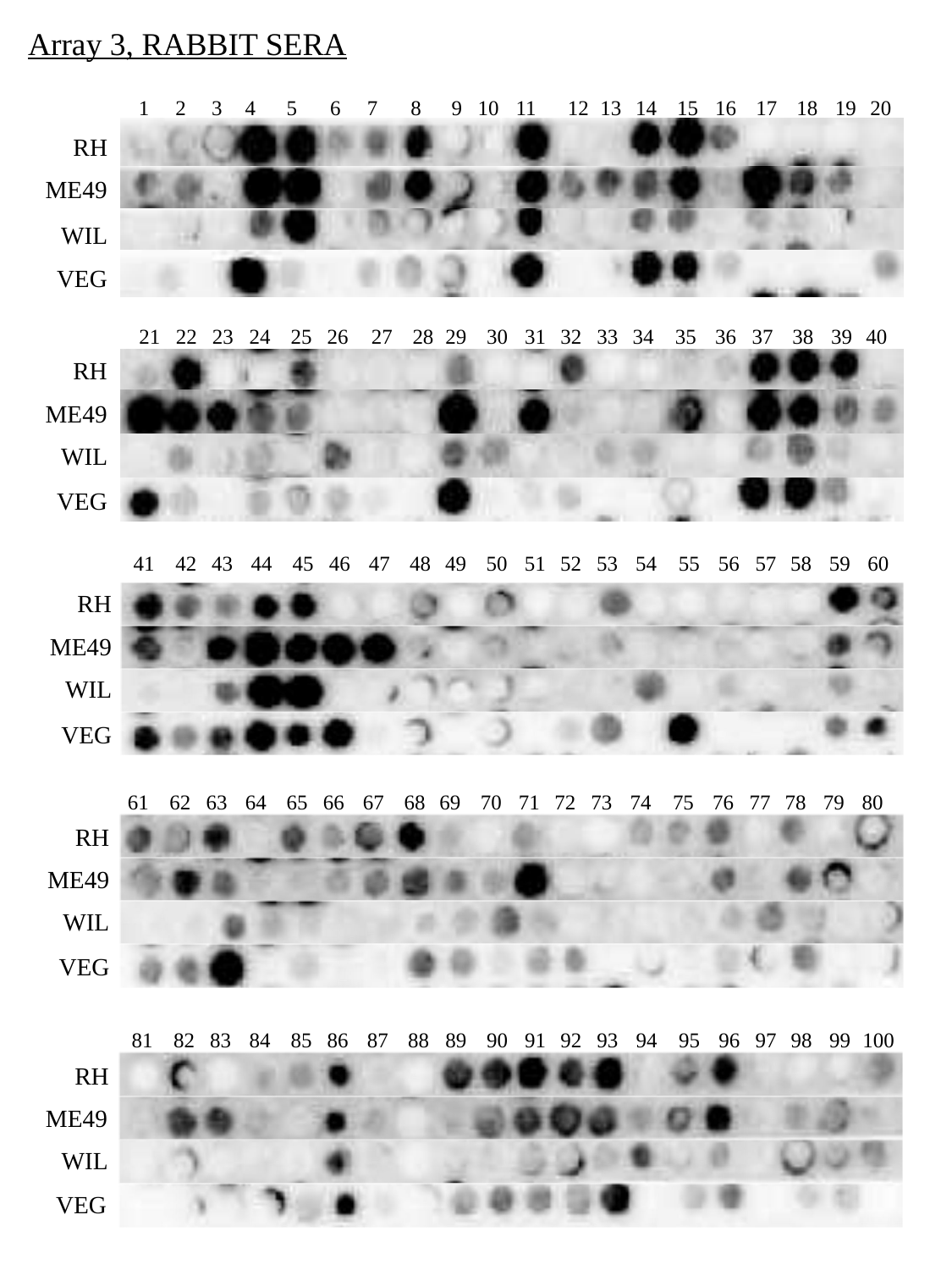

Array 3, RABBIT SERA
1
2
3
4
5
6
7
8
9
10
11
12
13
14
15
16
17
18
19
20
RH
ME49
WIL
VEG
21
22
23
24
25
26
27
28
29
30
31
32
33
34
35
36
37
38
39
40
RH
ME49
WIL
VEG
41
42
43
44
45
46
47
48
49
50
51
52
53
54
55
56
57
58
59
60
RH
ME49
WIL
VEG
61
62
63
64
65
66
67
68
69
70
71
72
73
74
75
76
77
78
79
80
RH
ME49
WIL
VEG
81
82
83
84
85
86
87
88
89
90
91
92
93
94
95
96
97
98
99
100
RH
ME49
WIL
VEG
