# Supplementary material for: Serotyping of Toxoplasma gondii Infection Using Peptide Membrane Arrays
Source: Front Cell Infect Microbiol. 2019 Nov 29;9:408. doi: 10.3389/fcimb.2019.00408 (PMC6895565; doi:10.3389/fcimb.2019.00408)
Supplement: Supplemental File 3 — Strips from array 4 comparing individual peptides for each serum sample from human patients. Strips from each array incubated with the different samples were taken and put together as a comparison. Peptide numbers are indicated above each group of strips. Patient identifications are indicated on the left side of each strip. Pt and Dn stand for Patient and Donor, respectively. [file Presentation_3.PPTX]

## Slide 1
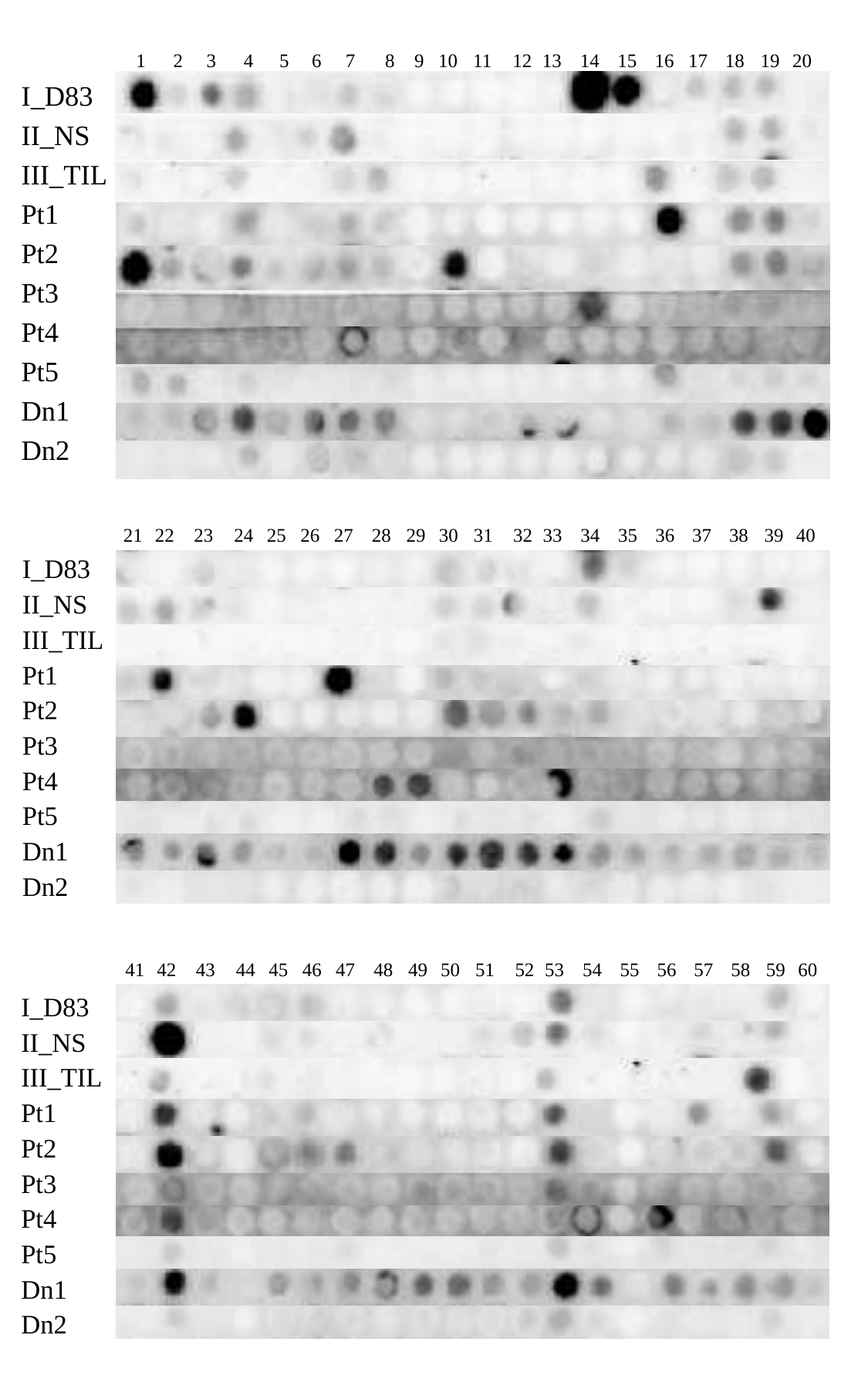

1
2
3
4
5
6
7
8
9
10
11
12
13
14
15
16
17
18
19
20
I_D83
II_NS
III_TIL
Pt1
Pt2
Pt3
Pt4
Pt5
Dn1
Dn2
21
22
23
24
25
26
27
28
29
30
31
32
33
34
35
36
37
38
39
40
I_D83
II_NS
III_TIL
Pt1
Pt2
Pt3
Pt4
Pt5
Dn1
Dn2
41
42
43
44
45
46
47
48
49
50
51
52
53
54
55
56
57
58
59
60
I_D83
II_NS
III_TIL
Pt1
Pt2
Pt3
Pt4
Pt5
Dn1
Dn2

## Slide 2
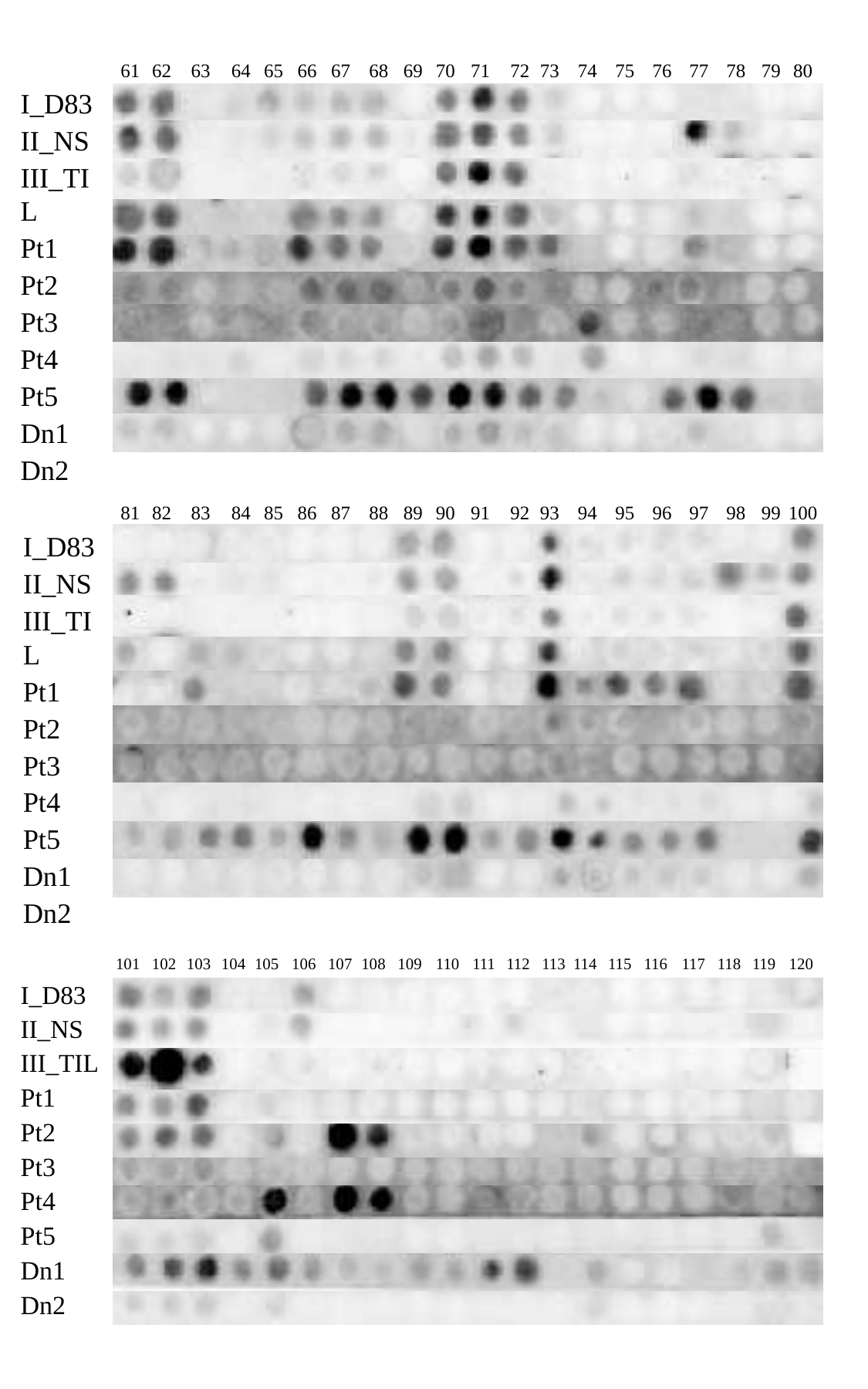

61
62
63
64
65
66
67
68
69
70
71
72
73
74
75
76
77
78
79
80
I_D83
II_NS
III_TIL
Pt1
Pt2
Pt3
Pt4
Pt5
Dn1
Dn2
81
82
83
84
85
86
87
88
89
90
91
92
93
94
95
96
97
98
99
100
I_D83
II_NS
III_TIL
Pt1
Pt2
Pt3
Pt4
Pt5
Dn1
Dn2
101
102
103
104
105
106
107
108
109
110
111
112
113
114
115
116
117
118
119
120
I_D83
II_NS
III_TIL
Pt1
Pt2
Pt3
Pt4
Pt5
Dn1
Dn2
