# Supplementary material for: Serotyping of Toxoplasma gondii Infection Using Peptide Membrane Arrays
Source: Front Cell Infect Microbiol. 2019 Nov 29;9:408. doi: 10.3389/fcimb.2019.00408 (PMC6895565; doi:10.3389/fcimb.2019.00408)
Supplement: Supplemental File 4 — Strips from array 5 comparing individual peptides for each serum sample from human patients. Strips from each array incubated with the different samples were taken and put together as a comparison. Peptide numbers are indicated above each group of strips. Patient identifications are indicated on the left side of each strip. [file Presentation_4.pptx]

## Slide 1
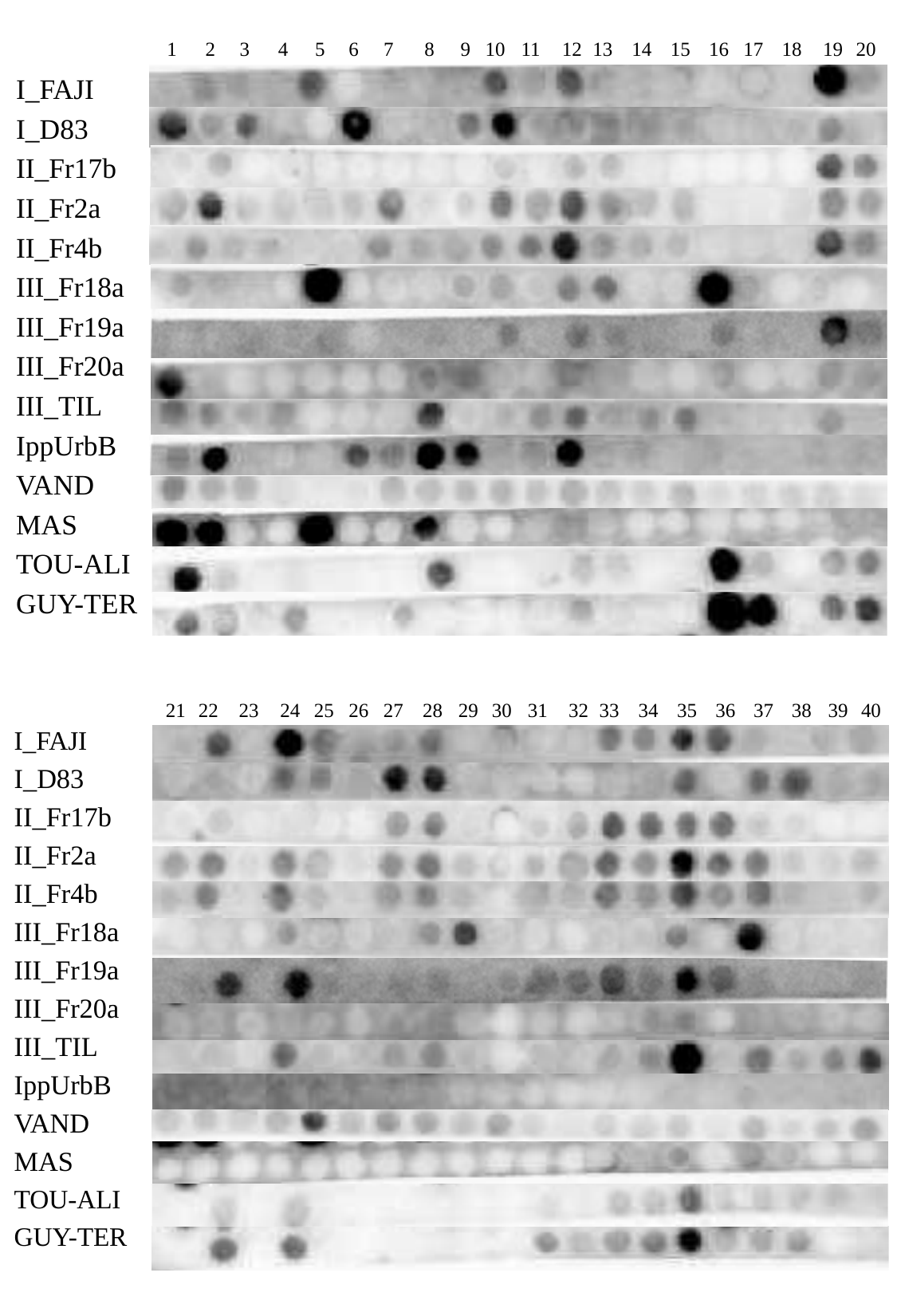

1
2
3
4
5
6
7
8
9
10
11
12
13
14
15
16
17
18
19
20
I_FAJI
I_D83
II_Fr17b
II_Fr2a
II_Fr4b
III_Fr18a
III_Fr19a
III_Fr20a
III_TIL
IppUrbB
VAND
MAS
TOU-ALI
GUY-TER
26
24
30
31
32
33
34
35
36
37
38
39
40
21
22
23
25
27
28
29
I_FAJI
I_D83
II_Fr17b
II_Fr2a
II_Fr4b
III_Fr18a
III_Fr19a
III_Fr20a
III_TIL
IppUrbB
VAND
MAS
TOU-ALI
GUY-TER
